# Supplementary material for: Identification and functional analysis of non-coding regulatory small RNA FenSr3 in Bacillus amyloliquefaciens LPB-18
Source: PeerJ. 2023 May 15;11:e15236. doi: 10.7717/peerj.15236 (PMC10194069; doi:10.7717/peerj.15236)
Supplement: Supplemental Information 4 [file peerj-11-15236-s004.zip › KO/CK-vs-T1_map/map01502.html]

KEGG PATHWAY: Vancomycin resistance - Reference pathway


|  |  |
| --- | --- |
| **Vancomycin resistance - Reference pathway** |  |

[
Pathway menu
| Organism menu
| Pathway entry
| Show description
| User data mapping
]

Vancomycin (VCM) is a glycopeptide antibiotic agent that inhibits the synthesis of peptidolgycan in the production of bacterial cell walls. It is active in most Gram-positive bacteria and acts by binding to the D-Ala-D-Ala C-terminus of pentapeptide, thus blocking the addition of subunits to the peptidoglycan backbone. The resistance to VCM was first described among enterococci in the late 1980s in Europe. Subsequently, vancomycin-resistant enteococci (VRE) have been observed in many countries. The genes encoding resistance are carried on plasmids that can tranfer themselves from cell to cell and on tranponsons that can jump from plamids to the chromosome. Up to now, at least six resistance operon types are characterized, namely VanA, VanB, VanC, VanD, VanE and VanG. The VanA and VanB operons are detected on plasmids or in the chromosome, whereas the rest are found in the chromosome. In addition, Streptomyces, which is non-pathogenic, non-glycopeptide-producing actinomycete, also shows high-level resistance to VCM. In Streptomyces, novel genes (vanK and vanJ) are characterized with no counterparts found in the resistance clusters of pathogenic bacteria.
The expression of vancomycin resistance pathway is induced by the VanS-VanR two component system, and D-Ala-D-Lac or D-Ala-D-Ser dipsipeptides are formed in place of D-Ala-D-Ala dipeptide. Thus, VCM cannot bind to pentadepsipeptide[D-Lac] or pentadepsipeptide[D-Ser]. The D-Ala-D-Lac variation shows high levels of resistance to VCM, especially in VanA type, wheras the D-Ala-D-Ser variation shows low levels of resistance. The modified pepidoglycan can interact with cross-linking enzymes (PBPs), thus cell wall is successfully formed.

|  |  |  |
| --- | --- | --- |
| Reference pathway | 184% 150% 122% 100% 82% 67% 55% | 图片下载 |
